# Correction: Verbesserung der Ernährungssituation von Patienten mit fortgeschrittenem nicht-kleinzelligem Lungenkarzinom (NSCLC) durch Off-label-Medikation

**DOI:** 10.1007/s00066-024-02314-7

**Published:** 2024-10-29

**Authors:** Daphne Schepers-von Ohlen

**Affiliations:** 1 Klinik für Strahlentherapie, UKSH, Campus Lübeck, Ratzeburger Allee 160, 23538 Lübeck, Deutschland; 2 Arbeitsgruppe junge DEGRO der Deutschen Gesellschaft für Radioonkologie e. V. (DEGRO), Berlin, Deutschland

**Correction:**

**Strahlenther Onkol 2024**

10.1007/s00066-024-02299-3

Der Titel dieses Artikels war fälschlicherweise mit „Verbesserung der Ernährungssituation von Patienten mit fortgeschrittenem kleinzelligem Lungenkarzinom (NSCLC) durch Off-label-Medikation“ angegeben. Richtig wäre „Verbesserung der Ernährungssituation von Patienten mit fortgeschrittenem nicht-kleinzelligem Lungenkarzinom (NSCLC) durch Off-label-Medikation“ gewesen.

Der Originalbeitrag wurde korrigiert.

The title of this article was incorrectly written as “Improving the nutritional situation of patients with advanced small cell lung cancer (NSCLC) using off-label medication”.

“Improving the nutritional situation of patients with advanced non-small cell lung cancer (NSCLC) through off-label medication” would have been correct.

The original article has been corrected.

